# Effect of Molecular Weight of Poly(vinyl alcohol) on Quercetin Release from Poly(vinyl alcohol) Films

**DOI:** 10.3390/polym18212759

**Published:** 2026-11-06

**Authors:** Caroline Beatriz de Oliveira, Nara Cristina de Souza, Paula Becker Pertuzatti Konda, Ricardo Stefani

**Affiliations:** 1 Programa de Pós-Graduação em Ciência de Materiais, Campus Universitário do Araguaia, Universidade Federal de Mato Grosso (UFMT), Barra do Garças 78605-091, MT, Brazil; 2 Laboratório de Nanosistemas e Tecnologias, Universidade Federal de Goiás (UFG), Ap. de Goiânia 74690-900, GO, Brazil; 3 Departamento de Química, Instituto de Ciências Exatas e Biológicas, Universidade Federal de Ouro Preto (UFOP), Ouro Preto 35400-000, MG, Brazil

**Keywords:** poly(vinyl alcohol), quercetin, active packaging, molecular weight, release kinetics, food simulants, antioxidant activity

## Abstract

Antioxidant active packaging delays lipid oxidation by transferring a bioactive compound from the polymer to the food, so the rate of that transfer is the property that matters. Poly(vinyl alcohol) (PVA) is a biodegradable, non-toxic film-former, but how its molecular weight governs polyphenol release has not been resolved. Films were cast from PVA of low (31–50 kDa), medium (85–124 kDa) and high (146–186 kDa) molecular weight in four formulations: unloaded (F1), quercetin pre-dissolved in ethanol at 0.5 (F2) and 1.0 g L^−1^ (F3), and quercetin added as a powder at 1.0 g L^−1^ (F4). The FT-IR of all four formulations showed the crystalline quercetin band set, led by the aromatic C=C mode at 1520 cm^−1^, only in F4; F3 carried the same loading and showed none of it, so the method of incorporation rather than the loading decides whether a crystalline quercetin phase forms. The X-ray diffraction of all four formulations confirmed a separate crystalline phase in F4 alone: the mean height of the four diagnostic quercetin reflections was 0.013–0.033 relative to the PVA (101) reflection in F1, F2 and F3 and 0.931 in F4, with the 12.54° reflection reaching 2.59–2.89 times the polymer reflection. Atomic force microscopy found both 1.0 g L^−1^ formulations rougher than the control at every molecular weight, regardless of whether a crystalline phase was present, so surface relief at this loading did not report crystallinity; the two differed in roughness from each other as much as from the control, so the method of incorporation affect topography as well. The antioxidant assays add the functional consequence of the structural difference: in the fatty-food simulant, the only medium in which quercetin was freely soluble, the dispersed form yielded 1.8 times the DPPH activity and 2.1 times the extractable phenolic content of the crystalline form at the same loading, while in the two aqueous simulants, they were indistinguishable. Quercetin release in PBS over 12 days, analysed as a mixed-effects model with a random intercept for each of the 26 specimens, was governed by a molecular weight × formulation interaction ($\chi^{2}=31.8$ on 4 d.f., p<0.0001) with neither main effect significant alone. F3 and F4 released statistically identical total amounts (28.4 and 29.2 μmol g^−1^, p=0.72) but in opposite molecular-weight orders (F(2,11)=6.61, p=0.013): the non-crystalline form released 15.9 μmol g^−1^ more from the low-molecular-weight matrix, the crystalline form 13.8 μmol g^−1^ more from the high-molecular-weight one. Across ABTS, DPPH and Folin–Ciocalteu assays, the extractable antioxidant was governed chiefly by the simulant, decreasing as ethanol 95% > ethanol 10% > acetic acid 3%, consistent with the poor aqueous solubility of quercetin, and was highest from the low-molecular-weight films in all three assays. Surface roughness also fell with molecular weight from $S_{q}=114.9$ to 30.3 nm in the unloaded films. The practical conclusion is that quercetin loading and the method of its incorporation must be chosen together with the polymer grade, because the incorporation method determines which molecular weight releases fastest.

## 1. Introduction

The food industry seeks ways to extend the shelf life of its products [1]. Active packaging, in which the package participates in preserving the food rather than acting only as a passive barrier, has become one of the most promising routes, particularly when built on biodegradable materials [2,3]. Because lipid oxidation is a dominant spoilage mechanism, most active films developed to date are antioxidant films. Poly(vinyl alcohol) (PVA) is an attractive matrix for this purpose: it is non-toxic, biocompatible, chemically stable, biodegradable, and an excellent film-former [4,5,6,7], and it has already been combined with natural antioxidant extracts to give functional packaging films [8,9].

Antioxidant packaging works by transferring the active compound from the polymer into the food, so the rate of that transfer, not merely the total antioxidant content, determines performance [10,11,12]. Both the chemistry of the antioxidant [13] and the composition of the matrix [14] shape this process. Quercetin [3,3′,4′,5,7-pentahydroxyflavone] is among the most studied flavonoids, combining well-documented antioxidant [15,16] and antimicrobial [17] activity with recognised health benefits [18], and it has been incorporated into a range of packaging matrices [19,20,21,22,23]. Most recent work on quercetin-loaded PVA films has pursued added complexity by blending PVA with chitosan, dispersing quercetin as self-assembled nanocrystals, or conjugating it to a second polymer to raise loading or retard release [24,25]. PVA molecular weight is known to set the chain entanglement density, free volume and degree of swelling of the matrix and control how a small molecule diffuses out of it [26,27,28]. Andrade et al. [29] showed that PVA molecular characteristics alter the structural and functional properties of films carrying phenolic acids but did not resolve how molecular weight acts on release once the antioxidant load is varied. The question matters practically, because loading and molecular weight are the two formulation levers available to a manufacturer and they may not act independently.

Therefore, this work evaluated PVA molecular weight as a design variable for quercetin release, crossing three molecular weights with two quercetin loadings and followed release over twelve days, so that molecular weight, loading and time, and their interactions, were separated statistically. Antioxidant activity was measured in three food simulants. Regulation (EU) No 10/2011 [30] prescribes different simulants for different food categories, and because quercetin is a poorly water-soluble flavonoid, its partitioning should depend strongly on the polarity of the contacting phase; the aqueous-acidic, aqueous-alcoholic and fatty-food simulants used here therefore bracket the range of conditions a packaging film would meet in service and allow the release results to be read as a function of food type [31].

## 2. Material and Methods

### 2.1. Material

Poly(vinyl alcohol) was obtained from Sigma-Aldrich (São Paulo, SP, Brazil) in three grades: low molecular weight (31,000–50,000 g mol^−1^, 98–99% hydrolysed), medium molecular weight (85,000–124,000 g mol^−1^, 99% hydrolysed) and high molecular weight (146,000–186,000 g mol^−1^, 99% hydrolysed). Quercetin hydrate (Sigma-Aldrich, São Paulo, SP, Brazil Art. No. 337951), absolute ethanol, Folin–Ciocalteu reagent, sodium carbonate, 2,2-diphenyl-1-picrylhydrazyl (DPPH), 2,2′-azino-bis(3-ethylbenzothiazoline-6-sulfonic acid) (ABTS), potassium persulfate, 6-hydroxy-2,5,7,8-tetramethylchroman-2-carboxylic acid (Trolox), glacial acetic acid and phosphate-buffered saline (PBS) were all of analytical grade. Distilled water was used throughout.

### 2.2. Film Preparation

Films were prepared by solvent casting. For each molecular weight, 1 g of PVA was dissolved in 100 mL of distilled water under magnetic stirring at 70 °C until the solution was clear, giving a 1% (*w*/*v*) film-forming solution that contained exactly 1 g of PVA. Quercetin loadings are therefore reported both as g of quercetin per liter of film-forming solution and as mg of quercetin per g of PVA.

Four formulations were cast from each molecular weight, differing in quercetin concentration and in how the flavonoid was introduced: **F1**, the control, received no quercetin; **F2**, 0.5 g L^−1^ (50 mg per g of PVA), with the quercetin pre-dissolved in 30 mL of ethanol before addition; **F3**, 1.0 g L^−1^ (100 mg g^−1^), likewise pre-dissolved in 30 mL of ethanol; and **F4**, 1.0 g L^−1^ (100 mg g^−1^), with the quercetin added directly as a powder.

All four formulations were cast from the same water–ethanol mixture. Each 100 mL aliquot of film-forming solution received 30 mL of ethanol: in F1, the ethanol was added without quercetin, in F2 and F3, the quercetin was dissolved in it before addition, and in F4, the same 30 mL of ethanol was added to the solution while the quercetin was introduced separately as a powder. Solvent composition, ethanol-to-water ratio and total cast volume were therefore identical across the series, and the only variable that distinguished F4 from F3 was whether the flavonoid entered the solution already in solution or as a solid. The suspensions were stirred until the flavonoid was fully dispersed. Each casting solution was divided among three acrylic Petri dishes (90 mm diameter) and dried in an oven at 35 °C for 24 h, until the solvent had completely evaporated and the mass was constant. One specimen was taken from each dried dish, so the three replicates of every formulation × molecular weight combination were films cast and dried separately from a common solution. Replication therefore spanned the casting and drying steps, in which film thickness, surface relief and solvent loss were determined.

Films are identified throughout by formulation and molecular weight—**F1L**, **F1M**, **F1H** for the unloaded controls cast from low-, medium- and high-molecular-weight PVA, and correspondingly **F2L–F2H**, **F3L–F3H** and **F4L–F4H**. FT-IR, X-ray diffraction and atomic force microscopy covered all four formulations; the release experiment covered the three loaded ones. Film thickness was measured with a digital micrometer at five randomly chosen points on each film and is reported as the mean of those five readings. One film per formulation × molecular weight combination was measured, so the five readings of each combination are positions within a single specimen and not independent replicate films; the replicate films described above were not individually gauged. Thickness was not equal across the series, ranging from 0.048 mm (F2H) to 0.128 mm (F1M), a 2.7-fold span. Because formulation and molecular weight were completely confounded with film identity in these measurements, there was no valid error term against which to test either factor, and the two-way analysis of variance reported in the previous version of this work was withdrawn in full; the variation among the series cannot be apportioned between formulation and molecular weight. For the record, the withdrawn test was computed on the 60 individual measurements and returned F(3,48)=19.29 for formulation, F(2,48)=17.10 for molecular weight and F(6,48)=10.57 for their interaction, all nominally p<0.0001; the 48 residual degrees of freedom cannot arise from twelve films. Thickness is given descriptively as the mean and standard deviation of the five positions of each film, and its relation to release is examined in Section 3.5 without an inferential test.

### 2.3. Film Characterisation

#### 2.3.1. FT-IR Spectroscopy

**Figure 1:**
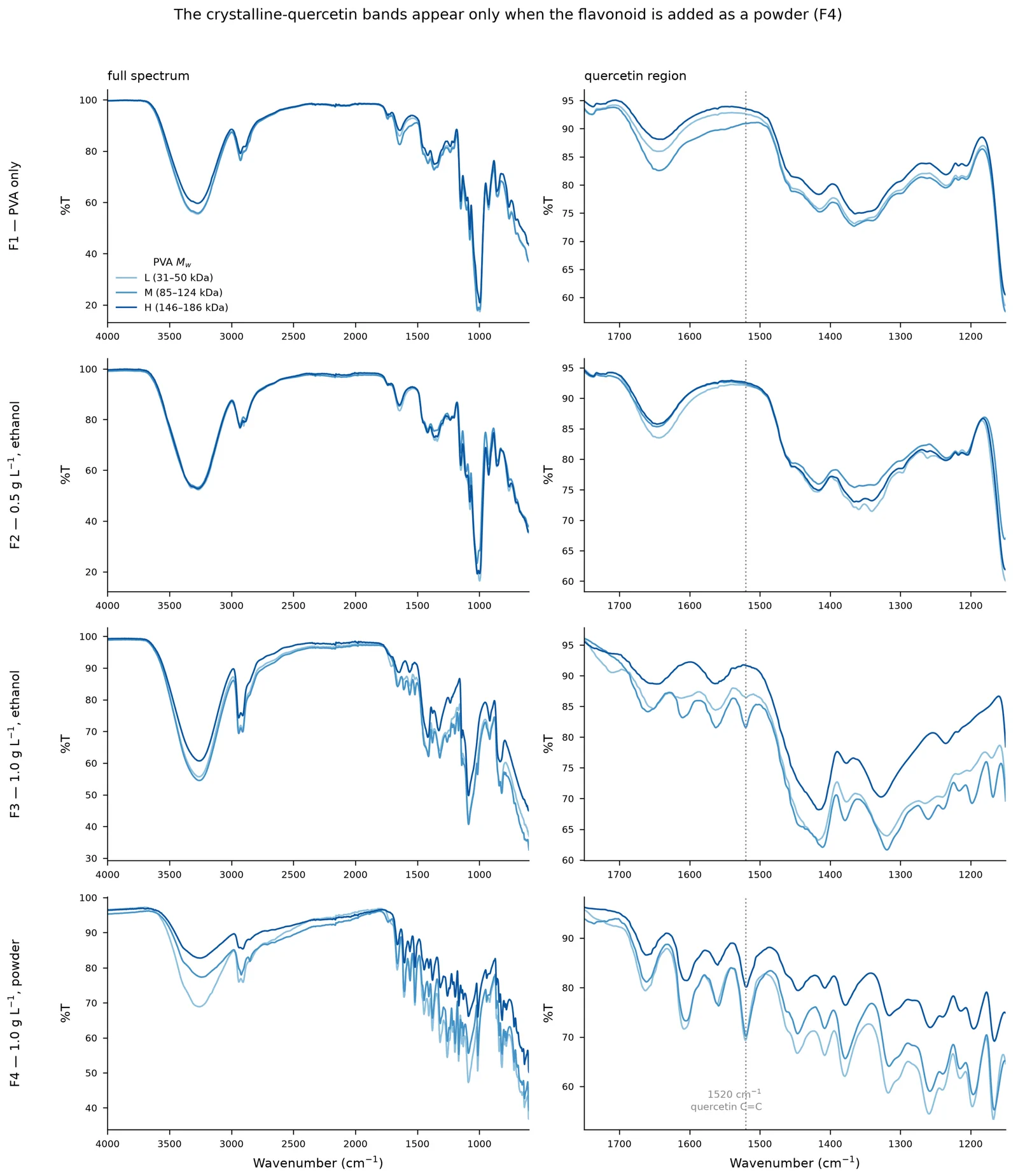
FT-IR spectra all films. Rows are formulations: F1 unloaded, F2 at 0.5 g L^−1^ and F3 at 1.0 g L^−1^ with quercetin pre-dissolved in ethanol, F4 at 1.0 g L^−1^ added as a powder; within each panel, the three traces are the low-, medium- and high-molecular-weight PVA. **Left** Full spectrum. **Right** The 1750–1150 cm^−1^ region, with the quercetin C=C band at 1520 cm^−1^ marked.

Spectra were recorded on a PerkinElmer Spectrum 100 (Perkin Elmer do Brasil, São Paulo, Brazil) spectrophotometer in attenuated total reflectance mode (diamond/ZnSe crystal) at 4 cm^−1^ resolution over 4000–600 cm^−1^. Spectra were recorded all twelve films over 4000–600 cm^−1^ with a 1 cm^−1^ data interval (3401 points per spectrum). All nine are plotted in Figure 1, grouped by formulation, and the band positions measured from the absorbance maxima of each spectrum are collected in Table 1.

#### 2.3.2. X-Ray Diffraction

Diffractograms were collected on a Bruker AXS D8 Advance diffractometer (Bruker AG, Karlsruhe, Germany) with a copper tube at 40 kV and 40 mA (Cu-Kα, λ = 1.5418 Å) over 4–50° (2θ). Specimens were cut flat and mounted on a zero-background holder. Scans were run in continuous θ–2θ coupled (Bragg–Brentano) reflection geometry at room temperature, 25 °C, in air, without sample spinning. The angular step was 0.050°.

#### 2.3.3. Atomic Force Microscopy

Surface topography was imaged on a Nanosurf EasyScan 2 microscope (NanoSurf AG, Liestal, Switzerland) with an EZ2-FlexAFM head and a Tap190Al-G cantilever, operating in intermittent-contact (phase-contrast) mode in air. Images of 512 × 512 pixels were acquired over 20 μm × 20 μm at 1 s per line. The images presented and analysed are the Z-axis topography channel of the forward scan; the amplitude and phase channels were recorded simultaneously. The procedure followed that previously described by our group for latex–propolis membranes [32].

Height data were exported from the native Nanosurf NID files and processed with a first-order plane subtraction to remove sample tilt. From the levelled height map z(x,y), we report the ISO 25178 [33] parameters $S_{a}$ (arithmetic mean height deviation), $S_{q}$ (root mean square height) and $S_{z}$ (maximum height range). One 20 μm image was acquired per formulation, so no between-image standard deviation was available; to quantify within-image heterogeneity, each image was divided into four non-overlapping 10 μm × 10 μm quadrants, each re-levelled independently, and the mean and standard deviation of $S_{q}$ across those quadrants are reported alongside the whole-image value. The entropy parameter *E* is the differential (Shannon) entropy of the height distribution evaluated under a Gaussian approximation, $E=\ln\left(S_{q}\right)+\frac{1}{2}\ln\left(2\pi e\right)$. *E* is a strictly monotonic function of $S_{q}$ and so conveys no information independent of the roughness. Although redundant at a first glance, *E* was introduced here due to its widespread use as a tool for texture image studies of biopolymer surfaces [34].

### 2.4. Antioxidant Activity

Three food simulants were prepared in accordance with Regulation (EU) No. 10/2011 [30]: S1, ethanol:water 10:90 (*v*/*v*), simulant A, for hydrophilic and alcoholic foods; S2, acetic acid:water 3:97 (*v*/*v*) for acidic foods; and S3, ethanol:water 95:5 (*v*/*v*). Simulants were designated S1–S3 to avoid any ambiguity with the film codes.

Film samples of 2 cm^2^ were immersed in 10 mL of each simulant in 100 mL beakers and sonicated for 10 min. Sonication was used to reach a near-equilibrium extract within a practical laboratory time, and it should be understood as an accelerated extraction rather than a simulation of service conditions; the reported values therefore represent the antioxidant capacity mobilisable from the film into each simulant and were expected to exceed what passive contact with a food would deliver over the same period. This is considered when interpreting the results in Section 3.4. Aliquots were withdrawn in 5 min intervals over 30 min. Table 2 reports the 30 min values, that is, the end point of the extraction at which the readings had stabilised.

The DPPH assay followed the method of Brand-Williams et al. [35], and the ABTS assay followed that described by Yang et al. [36], with absorbance read at 517 nm and 734 nm respectively after 30 min and 25 min of reaction. Total phenolic content was determined on the same extracts by the Folin–Ciocalteu method, with absorbance read at 740 nm, quantified against a gallic acid calibration curve and expressed as mg gallic acid equivalents (GAE) per 100 g of dry film. For each assay, a reagent blank consisting of the radical solution with the corresponding simulant replacing the sample was measured alongside the samples, so that any absorbance contributed by the simulant was subtracted; extracts of the unloaded F1 films in each simulant were measured as matrix controls and gave no detectable radical scavenging. Inhibition was calculated as $I\left(\%\right)=100\;\left(A_{\mathrm{blank}}-A_{\mathrm{sample}}\right)/A_{\mathrm{blank}}$ and converted to Trolox equivalents using a Trolox calibration curve (100–2000 μmol L^−1^) prepared in the same medium and run on the same day as the samples; results are expressed as μmol Trolox equivalents (TE) per g of dry film. All determinations were made in triplicate.

### 2.5. Quercetin Content and Release

The total reducing capacity of the films was determined with a Folin–Ciocalteu reagent [37]. The values are reported as total reducing capacity expressed in quercetin equivalents and were used only as a check that quercetin was incorporated in proportion to the amount added.

Quercetin release was quantified by UV–visible spectrophotometry at 429 nm on a PerkinElmer Lambda 25 instrument. The wavelength corresponds to the band I absorption maximum of quercetin, which is well-separated from PVA absorption and is the band conventionally used to quantify quercetin. A calibration curve was constructed from quercetin standards prepared in the same PBS used for the release experiment (pH 7.4) by dilution of a stock solution in ethanol such that the ethanol content of every standard and sample was identical and below 1% (*v*/*v*) over the range 10–200 μmol L^−1^ (six levels, each in triplicate); PBS containing the same ethanol fraction served as the blank. The calibration was A=0.0023C−0.0215 with $R^{2}=0.9949$. For the release experiment, square specimens of 0.5 × 0.5 cm, that is, an exposed area of 0.25 cm^2^ counting one face, were immersed in 3 mL of PBS in closed vials and held at 25 °C in the dark, without agitation, for 12 days. The medium was neither replaced nor sampled and replenished; the same 3 mL was read in place after 24, 96, 120, 192 and 288 h. So, each reading reports the quercetin present in the medium at that moment, and the values are not a running sum of interval increments. Concentrations were obtained from the calibration above and are reported as μmol per g of dry film, computed for each replicate individually from its own absorbance and its own specimen mass and then averaged, rather than by dividing a mean absorbance by a single specimen mass; specimen masses ranged from 1.2 to 5.6 mg across replicates. Three specimens were prepared for each of the nine formulation × molecular weight combinations and read at five times, giving 135 intended determinations, of which 127 were obtained. As one F3 medium-molecular-weight specimen was lost before the first reading and one F2 low-molecular-weight specimen had no readings at 24, 96 and 120, 8 of 135 intended readings were missing. Therefore, in order to not comprise the full analysis, the analysis used the 127 determinations actually measured, with no imputation. The chemical stability of quercetin in the release medium was not determined experimentally. The vials were closed and held in the dark, which removed photodegradation but not oxidation of the catechol B ring; quercetin is known to degrade in neutral and mildly alkaline aqueous media on a timescale of hours to days, depending on buffer composition, dissolved oxygen and co-existing solutes. Over a 12-day experiment, part of the quercetin that left the film was therefore expected to be lost from solution before it was read, and because the degradation products did not retain the band I chromophore, the absorbance at 429 nm reports the intact flavonoid rather than the total amount released [38,39]. No parallel stability control, and no separation of intact quercetin from its degradation products, was run alongside the release series, so this loss could not be quantified. The amounts in Table 3 are a lower bound on release and are treated throughout as a comparative rather than an absolute measure; the comparisons between formulations and molecular weights were unaffected to the extent that the degradation rate was common to all vials, which the identical medium, temperature and light conditions made the reasonable expectation but that was not verified.

### 2.6. Statistical Analysis

Formulation had three levels in the analyses, F2, F3 and F4; it was not equivalent to quercetin loading, because F3 and F4 carried the same nominal 1.0 g L^−1^ and differed in how the flavonoid was introduced, so only the F3–F4 contrast isolated the method. Molecular weight had three levels (low, medium, high), simulant three (S1–S3) and time five (24, 96, 120, 192 and 288 h). The unloaded control F1 carried no quercetin and was therefore excluded from every analysis of antioxidant activity and release.Antioxidant results were analysed as a three-way factorial design crossing formulation (3 levels) × molecular weight (3 levels) × simulant (3 levels), computed on the individual replicate values with all interaction terms; the extracts are independent samples, so a fixed-effects factorial model was appropriate there. Release results required a different model, because the same film specimen was returned to the spectrophotometer at all five times, and successive readings of one specimen were not independent. They were analysed as a linear mixed-effects model with formulation (3) × molecular weight (3) × time (5) as crossed fixed effects, all interactions included, and a random intercept for each of the 26 specimens, fitted by restricted maximum likelihood; fixed effects were tested by Wald tests on sum-to-zero contrasts, so that the terms reported are marginal effects, and nested fixed-effect structures were compared by likelihood-ratio tests on maximum-likelihood fits. Because formulation and molecular weight varied between specimens while time varied within them, the two strata had different precisions, and the simple effects of molecular weight within each formulation were therefore tested on the specimen means, that is, on one value per specimen averaged over the time course, which gave 26 experimental units and exact degrees of freedom. Because one specimen contributed two readings rather than five, specimen means and observation means coincide in eight of the nine combinations and differ in the ninth: F2 at low molecular weight averages 22.6 μmol g^−1^ over its 12 observations and 24.0 over its three specimens. The tables and the descriptive text report observation means, the tests used specimen means, and the two are distinguished wherever the difference arises.

Analyses were performed in Python 3.11 with statsmodels 0.14.

## 3. Results and Discussion

### 3.1. FT-IR Spectroscopy

Figure 1 shows the FT-IR spectra of all films, grouped by formulation, and Table 1 lists the band positions. All films display the bands characteristic of PVA: a broad O–H stretching envelope near 3270 cm^−1^, C–H stretching at 2906–2940 cm^−1^, a band near 1640–1660 cm^−1^, CH_2_ bending at 1406–1417 cm^−1^, C–H wagging at 1315–1345 cm^−1^, C–O stretching at 1234–1275 cm^−1^ and the (C–O)–C–OH mode at 1074–1088 cm^−1^. The compositional marker is the aromatic C=C band of quercetin at 1520 cm^−1^ [40], and it discriminated sharply between the formulations. Measured as depth above a local baseline interpolated between 1560 and 1480 cm^−1^, it was negative. That is, no band was resolved in every film of F1 (−0.010 absorbance units, averaged over the three molecular weights), F2 (−0.012) and F3 (−0.013), and positive in every film of F4 (+0.047). In the right-hand panels in Figure 1, the first three rows are smooth through this region, while the F4 spectra resolve a dense series of sharp bands. F3 and F4 contained the same quercetin loading. Pre-dissolving the flavonoid in ethanol before casting (F3) left no spectroscopic trace of crystalline quercetin, whereas adding the same quantity as a powder (F4) produced the full crystalline band set. The method of incorporation determined whether the flavonoid stayed in a non-crystalline state or as a separate crystalline phase, which is supported by diffraction data in Section 3.2.

The O–H region shows a smaller signal. Averaged over the three molecular weights, the O–H maximum moves from 3271 cm^−1^ in the unloaded control to 3270 cm^−1^ in F2, 3260 cm^−1^ in F3 and 3253 cm^−1^ in F4. The shift is in the direction expected if the phenolic hydroxyls of quercetin form additional hydrogen bonds with the hydroxyls of PVA, lengthening and weakening the O–H bond, and it scales with the quercetin content of the ethanol-dissolved series: negligible at 0.5 g L^−1^ and 11 cm^−1^ at 1.0 g L^−1^. The band near 1640–1660 cm^−1^ moves in step, from 1638–1645 cm^−1^ in F1 to 1646–1659 cm^−1^ in F3 and 1660–1662 cm^−1^ in F4, and the C–O stretch from 1234 to 1259–1275 cm^−1^. These results are in accordance for assignments for flavonoid-containing films reported in the literature [41].

### 3.2. X-Ray Diffraction

**Figure 2:**
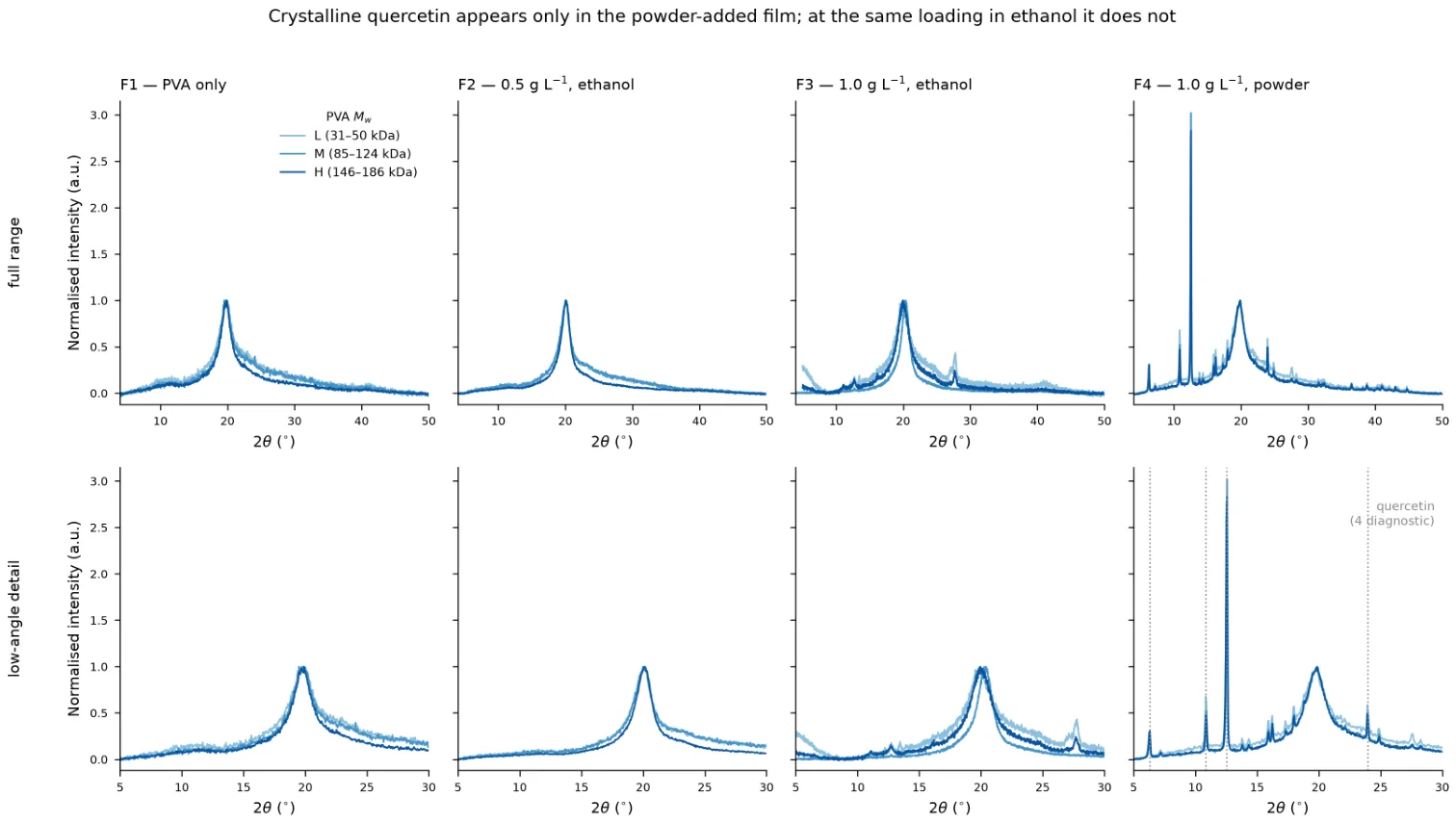
X-ray diffractograms of all films. Top row: full range. Bottom row: low-angle detail on a common scale.

Figure 2 shows the diffractograms of all films, normalised to the height of the PVA reflection and superimposed within each formulation. All samples exhibit the broad reflection near 19.5–20.4° (2θ) characteristic of semi-crystalline PVA plane [42], superimposed on a large amorphous halo, confirming that the films were predominantly amorphous with limited crystalline order.

The reflection narrowed as molecular weight rises in the control and in both formulations measured in the same session: its full width at half-maximum fell from 3.32 to 2.62 to 2.06° across F1L, F1M and F1H, from 2.11 to 2.16 to 1.76° across F2 and from 3.22 to 2.21 to 2.16° across F4. From the Scherrer equation, these correspond to apparent crystallite sizes growing from 2.4 to 3.9 nm, 3.8 to 4.6 nm and 2.5 to 3.7 nm respectively, and the crystallinity index rises in parallel (31.1, 33.0 and 41.8% for F1; 37.7, 36.8 and 49.2% for F2; 32.6, 39.0 and 39.9% for F4). Longer chains therefore develop more, and larger, crystalline domains on casting, irrespective of the quercetin content. The diffractograms of F4 films contain sharp reflections whose positions correspond to crystalline quercetin, and these are absent from F1, F2 and F3 alike. Of the nine quercetin positions examined, only four laid where PVA did not scatter and were therefore diagnostic: 6.31, 10.86, 12.54 and 23.95°. Expressed as height above the local background relative to the PVA reflection, their mean was 0.026 in F1, 0.013 in F2 and 0.033 in F3, against 0.931 in F4. The reflection at 12.54° alone reaches 2.59 to 2.89 times the height of the polymer reflection in F4, against a maximum of 0.071 anywhere else. The remaining five quercetin positions (13.78, 15.90, 16.24, 24.86 and 27.57°) coincide with weak PVA features; they are reported in Table 4. Because F3 and F4 contained the same 1.0 g L^−1^ loading and, as set out in Section 2.2, were cast from the same water–ethanol mixture at the same total volume, the solvent composition is not a competing explanation for the difference between them: the crystalline phase is produced by adding the flavonoid as a powder, not by exceeding a solubility limit at the higher loading.

As X-ray diffraction is insensitive to amorphous material and to crystallites below roughly 3–5 nm, the absence of quercetin reflections in F2 and F3 established only that no detectable crystalline quercetin phase was present. This indicated an amorphous solid dispersion [43,44], which is consistent with molecular dispersion but equally consistent with amorphous domains or aggregates of the flavonoid. Where F2 and F3 are described below as carrying the flavonoid in a “dispersed” or “non-crystalline” state, the term is shorthand for the experimental observation that no crystalline quercetin phase was detectable; the F3–F4 contrast is accordingly a contrast between a non-crystalline and a crystalline state, whatever the organisation of the former proves to be.

### 3.3. Atomic Force Microscopy

**Figure 3:**
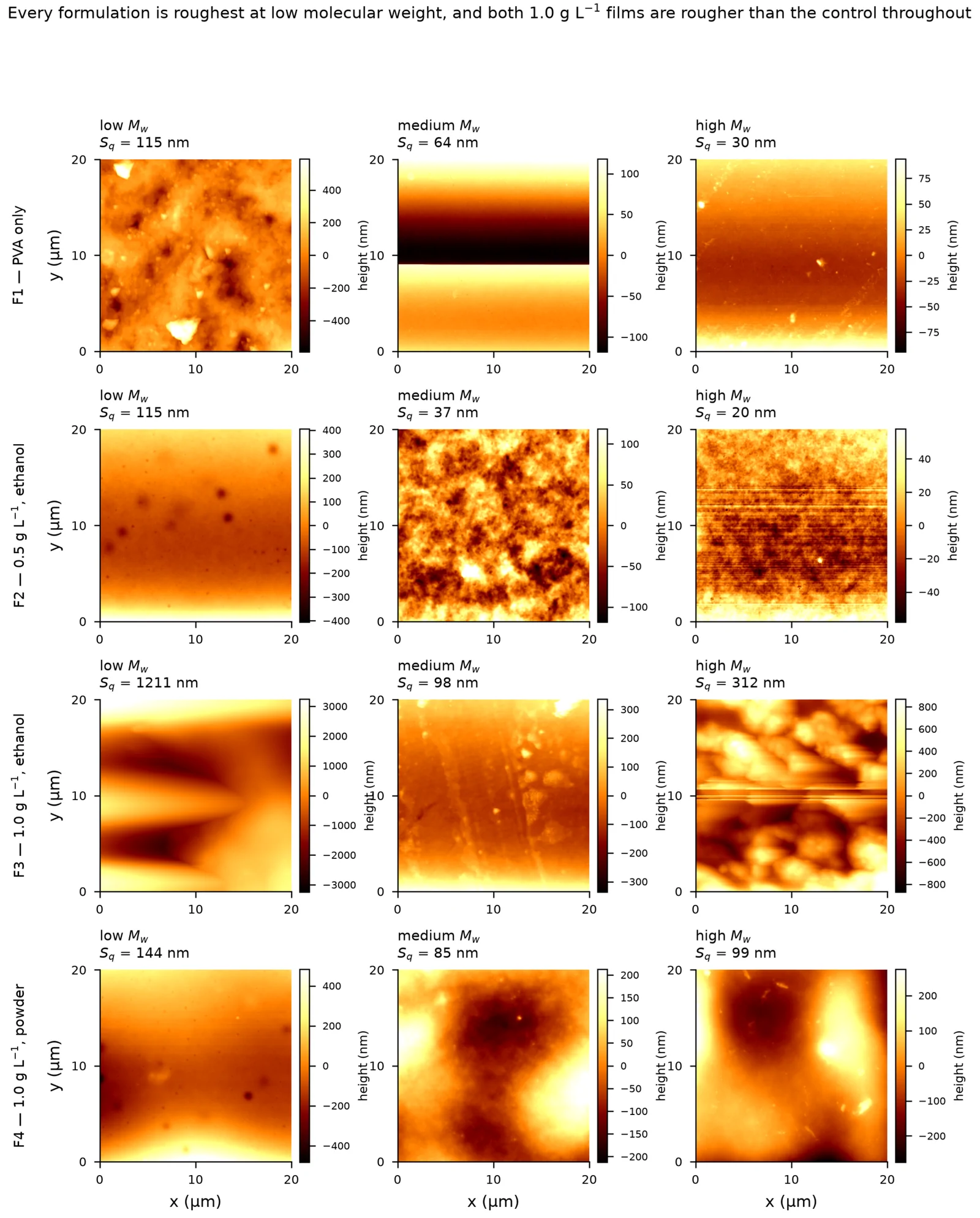
AFM topography (Z-axis channel, forward scan, intermittent-contact mode) of all films over 20 μm × 20 μm. Rows are formulations, columns are low-, medium- and high-molecular-weight PVA.

Figure 3 shows the Z-axis topography of all films over 20 μm × 20 μm, and Table 5 lists the corresponding roughness parameters.

From Figure 3, one can see that every formulation ws roughest at low molecular weight: $S_{q}$ was 114.9 nm in F1L against 64.1 and 30.3 nm for the medium- and high-molecular-weight controls, 114.5 against 37.4 and 20.3 nm across F2, 1211 against 97.5 and 312.4 nm across F3, and 143.7 against 85.4 and 99.2 nm across F4. The decrease was not monotonic, however, and should not be described as such: only in F1 and F2 did $S_{q}$ fall at each successive molecular weight. In F3 and F4, the high-molecular-weight film was rougher than the medium-molecular-weight one—312.4 against 97.5 nm in F3 and 99.2 against 85.4 nm in F4—so what the data support is that the low-molecular-weight film was the roughest of each series, not that roughness declines steadily with molecular weight. Higher-molecular-weight solutions are more viscous and their chains more entangled, which suppresses convective flow and surface-tension-driven relief during the slow evaporation of the casting solvent, producing a flatter surface; the same trend was reported for PVA films whose surface morphology was followed by AFM as a function of composition [45]. Both 1.0 g L^−1^ formulations were rougher than the control at every molecular weight, whereas F2 at half that loading was as smooth as the control or smoother. Quercetin at the higher loading therefore roughened these surfaces whichever way it was introduced. F3 and F4 carrie the same nominal loading but differed in $S_{q}$ by factors of 8.4, 1.1 and 3.1 at low, medium and high molecular weight, which is the same order as the difference between either of them and the control. The method of incorporation evidently affects surface topography as well; what the data do not establish the direction, since a single image per film gives no uncertainty against which to judge these ratios. The $S_{q}$ values of the four quadrants of each image (Table 5) differ considerably from one another, for instance, 44.4 ± 31.6 nm for F2L and 828.9 ± 424.6 nm for F3L, showing that these surfaces are heterogeneous on the 10 μm scale [46]. The F1M scan contains a horizontal band arising from a momentary loss of feedback during acquisition, which inflates its whole-image $S_{q}$ relative to the quadrant mean (64.1 nm against 28.2 ± 22.0 nm); the F1M roughness should therefore be treated as an upper bound. Small, roughly circular depressions a few hundred nanometres across were visible on several surfaces, most of them on F1L and F2L. They were shallow relative to the overall height range and were most plausibly residual solvent evaporation pits formed where vapour escaped through the drying skin, a common feature of solution-cast films dried without humidity control. They were shallow relative to the overall height range of the same images a few tens of nanometres against an $S_{z}$ of several hundred, so they were surface features rather than openings through a film some 0.1 mm thick; establishing that none of them penetrated further would have required a cross-sectional examination, which was not performed.

### 3.4. Antioxidant Activity

**Figure 4:**
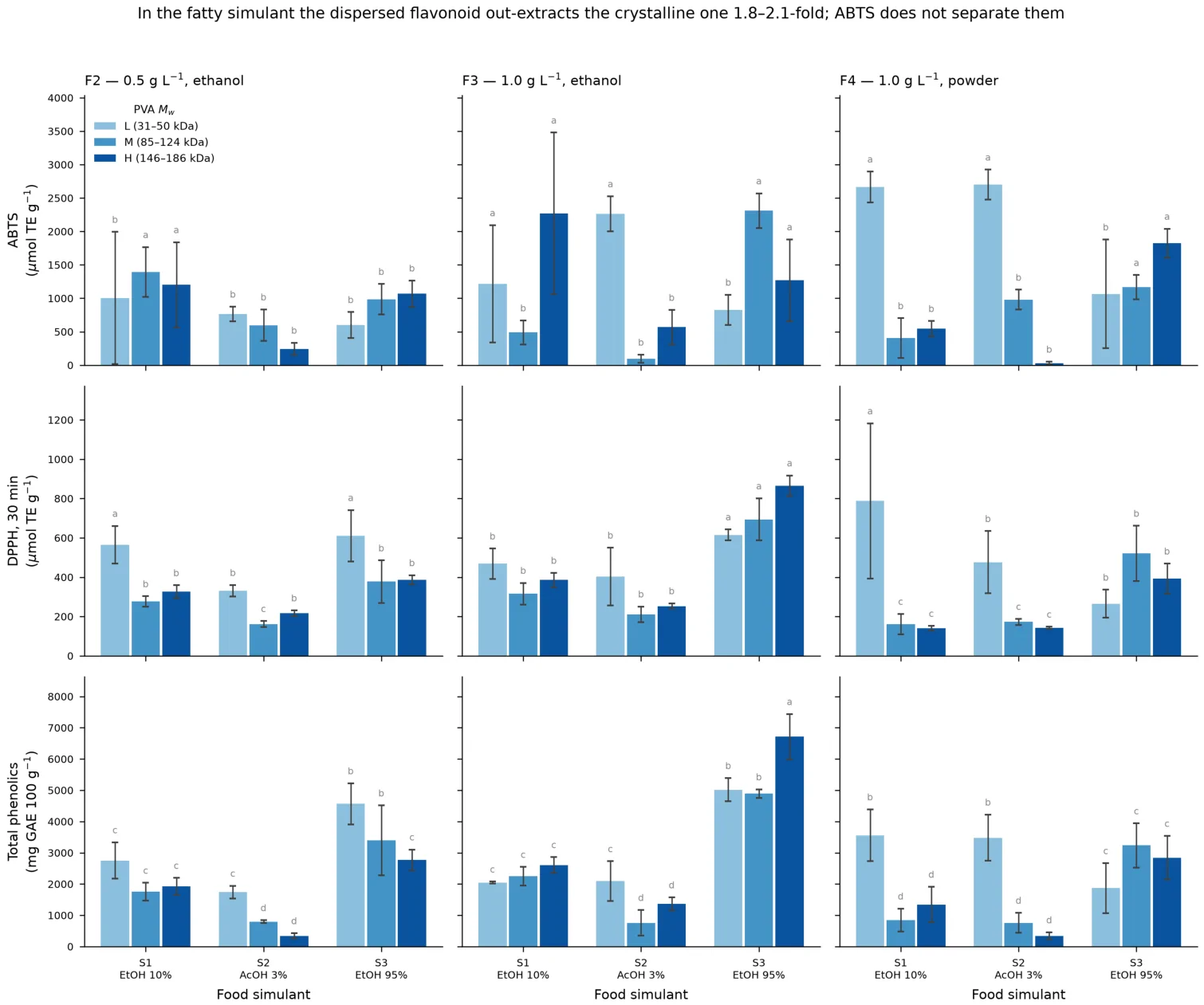
ABTS radical scavenging (**top**), DPPH radical scavenging at 30 min (**middle**) and total phenolic content by Folin–Ciocalteu (**bottom**) of the three loaded formulations after 30 min of contact with each food simulant. F2, 0.5 g L^−1^ pre-dissolved in ethanol; F3, 1.0 g L^−1^ pre-dissolved in ethanol; F4, the same 1.0 g L^−1^ added as a powder. S1, ethanol:water 10:90; S2, acetic acid:water 3:97; S3, ethanol:water 95:5. Bars are means of three separately cast films and error bars one standard deviation; letters denote Tukey groupings across all 27 combinations within each assay (α=0.05).

Table 2 reports the ABTS and DPPH radical scavenging activity and the total phenolic content of the loaded films in the three simulants, and Figure 4 displays the same data. In all three assays, every main effect and almost every interaction was significant, including the three-way interaction (ABTS F=6.11, DPPH F=4.62, total phenolics F=7.57; all p<0.001). The main effects are therefore reported as averages, and the formulation contrasts were tested as simple effects within each simulant. For ABTS, the effects were formulation F=4.64 (p=0.014), molecular weight F=7.40 (p=0.0016) and simulant F=3.86 (p=0.028); for DPPH, F=11.3, 23.2 and 41.3, respectively; and for total phenolics, F=31.7, 25.1 and 182.4 (all p<0.0001). Averaged over all films, DPPH activity decreased in the order S3 (ethanol 95%, 525.3 μmol TE g^−1^) > S1 (ethanol 10%, 380.7) > S2 (acetic acid 3%, 263.1), and total phenolics gave the same ranking with a much larger spread (3923, 2120 and 1297 mg GAE 100 g^−1^). The fatty-food simulant extracted the most antioxidant. This followed from the solubility of quercetin, which is sparingly soluble in water but freely soluble in ethanol, so the 95% ethanol medium both dissolved the flavonoid efficiently and swelled the PVA matrix, accelerating its egress; swelling of the polymer by the contacting phase is well-established as a promoter of migration in fatty-food simulants [47,48]. The acidic simulant, almost entirely aqueous, extracted the least in all three assays. ABTS was an exception to the ranking as S1 (1243 μmol TE g^−1^) was marginally above S3 (1215) and separated neither from the other, which is consistent with the greater dispersion of that assay discussed below.

Molecular weight acted in the opposite direction to its effect on release, discussed below, and did so consistently in all three assays, as the low-molecular-weight films yielded the most extractable antioxidant, while the medium and high grades were lower and indistinguishable from each other (DPPH 502.5, 321.3 and 345.3 μmol TE g^−1^ for low, medium and high, with low differing from both of the others at p≤0.015 and medium from high at p=0.90; total phenolics 3013, 2077 and 2251 mg GAE 100 g^−1^; ABTS 1435, 927 and 1008 μmol TE g^−1^). The effect was therefore a step between the lowest grade and the rest. Over a 30 min sonicated extraction, we measured how readily quercetin could be removed from the matrix, and the denser, more entangled higher-molecular-weight films resisted that removal; this is consistent with their higher crystallinity and greater chain entanglement [49]. Comparing F3 against F4, the dispersed form delivered more extractable antioxidant in two of the three assays DPPH 468.0 against 339.8 μmol TE g^−1^ (F=15.2, p=0.0004) and total phenolics 3083 against 2031 mg GAE 100 g^−1^ (F=55.2, p<0.0001), while ABTS found them equal (1257 against 1236, F=0.03, p=0.86). The difference was not uniform across media; it was confined almost entirely to the fatty simulant, where the dispersed form out-extracted the crystalline one 1.8-fold by DPPH (724.7 against 393.0; F=65.2, p<0.0001) and 2.1-fold by total phenolics (5542 against 2648; F=98.2, p<0.0001), whereas in S1 and S2, the two formulations were statistically indistinguishable in both assays (p=0.10 to 0.74). The 95% ethanol was the only one of the three media in which quercetin was freely soluble, so it was the only one in which the dissolution of crystallites, rather than the solubility ceiling of the medium, limited what could be extracted. In the two aqueous simulants both formulations were held to the same low ceiling regardless of the state of the flavonoid within the film. The antioxidant assays therefore corroborate, from a wholly independent measurement, the conclusion drawn in Section 3.1 and Section 3.2 that pre-dissolution in ethanol leaves the quercetin in a non-crystalline state, whereas direct addition of the powder leaves a crystalline phase, and they added the functional consequence: the dispersed form is the more available one but only in a medium that can dissolve it.

### 3.5. Quercetin Release

**Figure 5:**
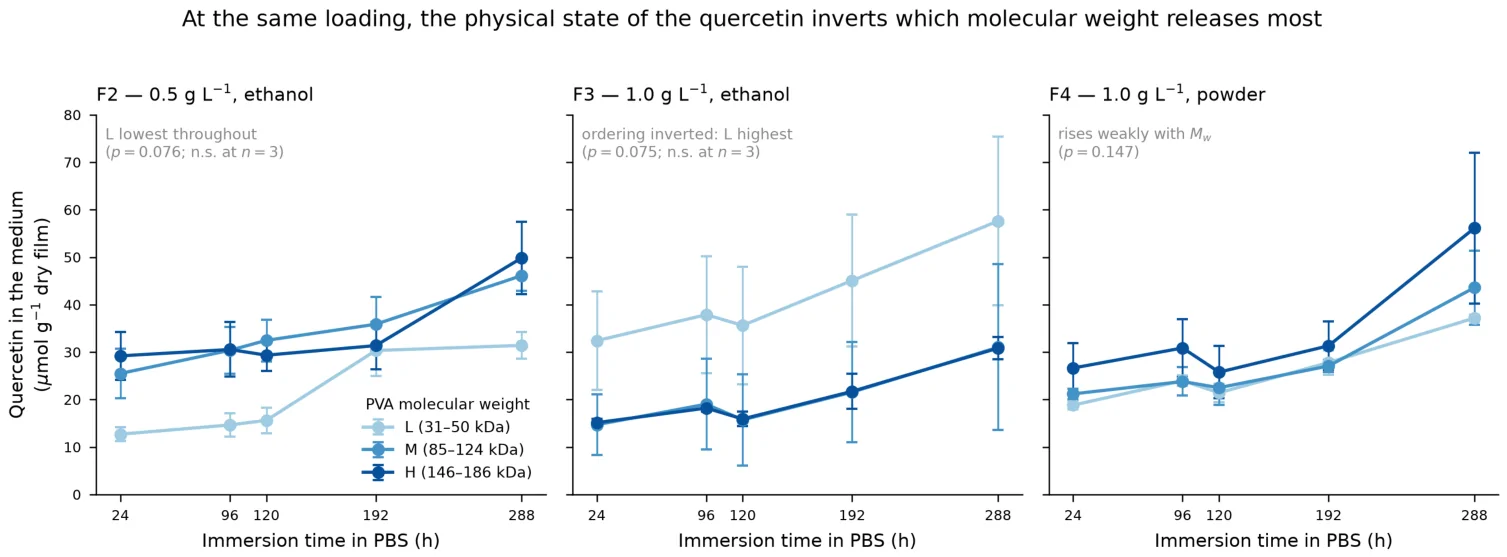
Cumulative quercetin released into PBS (pH 7.4, 25 °C, dark, static) over 12 days from F2 (0.5 g L^−1^, ethanol), F3 (1.0 g L^−1^, ethanol) and F4 (1.0 g L^−1^, powder). Points are means of three separately cast films and error bars one standard deviation; each replicate was quantified from its own absorbance and its own specimen mass.

Table 3 reports the quercetin present in the PBS over 12 days, and Figure 5 shows the corresponding profiles. Release was followed for the three loaded formulations: F2, F3 and F4. Because the medium was never replaced, each value is the amount in solution at that moment, obtained from a single absorbance of that medium, and not a running sum of interval increments; it is therefore the net result of release into the medium and of any loss from it. Release rises with time in every formulation, as expected for diffusion from a loaded matrix, and the profiles are comparable in shape to those reported for polyphenols leaving PVA-based active films [50]. Because the same specimen was returned to the spectrophotometer all five times, the five readings of one specimen are repeated measures on a single experimental unit and the release data were analysed with the mixed-effects model described in Section 2.6, with a random intercept for each of the 26 specimens. The correction was not cosmetic: the between-specimen variance component was 39.4 (μmol g^−1^)^2^ against a within-specimen residual of 9.2, an intraclass correlation of 0.81, so four-fifths of the variance in the data was between specimens rather than within them.

The formulation × molecular weight interaction was the dominant effect between specimens (Wald $\chi^{2}=31.79$ on 4 d.f., p<0.0001; equivalently F(4,17)=6.53, p=0.0023 on the 26 specimen means), and neither main effect approached significance on its own (formulation $\chi^{2}=0.49$ on 2 d.f., p=0.78; molecular weight $\chi^{2}=0.57$ on 2 d.f., p=0.75). This combination—a large interaction with no main effects—is the signature of levels that pull in opposite directions and cancel when pooled. Time was highly significant ($\chi^{2}=752.9$ on 4 d.f., p<0.0001) and so were all of its interactions: formulation × time $\chi^{2}=19.40$ on 8 d.f., p=0.013; molecular weight × time $\chi^{2}=32.56$ on 8 d.f., p<0.0001; three-way $\chi^{2}=41.08$ on 16 d.f., p=0.0005. Likelihood-ratio tests on nested fixed-effect structures agreed ($\chi^{2}=49.4$ on 16 d.f. for the three-way term; $\chi^{2}=88.8$ on 32 d.f. for all time interactions jointly, both p<0.0001). The profiles therefore differed in shape as well as in level, and the ordering of the films was not fixed within the first 24 h.

Decomposing the interaction by formulation shows that molecular weight matters in each, although not in the same direction, but that, at three specimens per combination, none of the three simple effects reached significance when tested on the specimen means: F(2,6)=4.10, p=0.076 for F2, F(2,5)=4.54, p=0.075 for F3 and F(2,6)=2.69, p=0.147 for F4, with no Tukey contrast surviving adjustment in any of them. The orderings below are therefore descriptive patterns, anchored in a significant interaction, rather than individually demonstrated simple effects. In F2, the low-molecular-weight film released the least: 22.6 μmol g^−1^ averaged over the time course, against 34.1 for both medium and high. In F3, the ordering was inverted, the low-molecular-weight film releasing the most, 41.7 μmol g^−1^ against 20.4 for both medium and high. In F4, release increased weakly with molecular weight (25.8, 27.6 and 34.2 μmol g^−1^). At 288 h, the three series read 31.4, 46.1 and 49.8 μmol g^−1^ for F2; 57.6, 31.0 and 30.8 for F3; and 37.2, 43.6 and 56.1 for F4. It is the contrast between the F2 and F3 orderings, and between those and F4, that the interaction term certifies.

When comparing F3 against F4, which contained the same 1.0 g L^−1^ loading and differed only in whether the quercetin was pre-dissolved in ethanol, it was seen that they released statistically identical amounts, 28.4 and 29.2 μmol g^−1^, formulation effect F(1,11)=0.13, p=0.72, yet their interaction with molecular weight was significant (F(2,11)=6.61, p=0.013), and the two orderings were opposed. Within the high-molecular-weight matrix, the crystalline form released 13.8 μmol g^−1^ more than the non-crystalline one, a difference that was significant on the specimen means (p=0.031); within the low-molecular-weight matrix, the non-crystalline form released 15.9 μmol g^−1^ more, a difference of comparable size and opposite sign, which, with three specimens, fell short of significance on its own (p=0.108) and rested on the interaction term. The physical state of the flavonoid therefore did not change how much was released in total; the evidence shows that the interaction changed which polymer was released, not the pair of simple contrasts. In Table 3, between 96 and 120 h, the measured amount falls in seven of the nine combinations, which a quantity described as cumulative could not do. Two things account for this. As set out in Section 2.5, the medium was never replaced, so what is tabulated is the quercetin present in the solution at each time, computed independently from that time’s absorbance. Therefore, the measured quantity is release minus whatever leaves solution, as quercetin is appreciably unstable in neutral buffer, and the 429 nm absorption is lost as it degrades [38,39]. Furthermore, a loss mechanism acting continuously would depress every interval, but averaged over the nine combinations, the net rate was −0.069 μmol g^−1^ h^−1^ between 96 and 120 h against +0.051, +0.089 and +0.129 μmol g^−1^ h^−1^ for the intervals 24–96, 120–192 and 192–288 h, and no combination fell in any interval other than 96–120 h. A decline confined to one reading and shared by almost every sample measured that day points to a systematic deviation in that reading session rather than to a property of the films.

Refitting the mixed model to the remaining four times, the formulation × molecular weight interaction remained the dominant effect ($\chi^{2}=30.36$ on 4 d.f., p<0.0001, against $\chi^{2}=31.79$ all five times; F(4,17)=6.53, p=0.0022 on the specimen means, against p=0.0023); neither main effect was significant (formulation p=0.81, molecular weight p=0.67), the time interactions remained significant, and the descriptive orderings kept their signs: the low-molecular-weight film still released the least in F2 (24.0 against 34.5 and 35.2 μmol g^−1^), the ordering was still inverted in F3 (43.2 against 21.6 and 21.5), and release still rose weakly with molecular weight in F4 (26.9, 28.9, 36.2). The 120 h reading affected none of the comparisons on which this work rests.

When quercetin is non-crystalline (F3), release requires diffusion through the polymer, and the least entangled, most readily swollen low-molecular-weight matrix offers the least resistance [51]; hence, the low-molecular-weight film released the most. When a separate crystalline phase is present (F4), release requires that phase to dissolve, the crystallites must dissolve before the flavonoid can leave, and that step is largely independent of the matrix around them; surface-sited crystals are accessible to the medium irrespective of the matrix beneath, so the molecular-weight dependence weakens, and what remains favours the higher molecular weights whose smoother surfaces contain the most exposed crystalline material. F2 then fell into place as the dispersed case at half the loading, where the low-molecular-weight film released the least because its much rougher surface ($S_{q}=114.5$ nm against 37.4 and 20.3 nm) favoured the local aggregation of the small amount of flavonoid present.

Film thickness varied across the series and in principle governs release. The twelve films spanned 0.048 to 0.128 mm in mean thickness, a 2.7-fold range (Table 6), and the pattern was not a simple function of either factor: the thickest film was the medium-molecular-weight control and the thinnest the high-molecular-weight F2. As explained in Section 2.2, only one film per combination was gauged, so this variation could not be tested statistically and is reported as observed. A thicker film presents a longer diffusion path, a smaller surface-to-volume ratio and a larger reservoir to hydrate before the flavonoid can leave, so each of these should slow release and flatten its early profile. Across the nine formulation × molecular weight combinations followed here, however, thickness did not account for what was observed: it was uncorrelated with release, both with the time-averaged amount (Pearson r=−0.04, p=0.92; Spearman ρ=−0.02) and with the 288 h amount (r=−0.04, p=0.91). The two films that released the most, F3 at low molecular weight and F4 at high, were among the thickest and the thinnest respectively (0.126 and 0.080 mm), and the thinnest film of the series, F2 at high molecular weight (0.048 mm), was not the fastest releaser. That absence of correlation should not be read as evidence that thickness is not important. With nine points, only a strong monotonic relation was detectable (|r|≥0.67 at α=0.05); the thickness values themselves carried no replication, being single films measured at five positions, so the correlation was computed between nine cell means and nine single-film thicknesses and has no error structure on either axis. More fundamentally, thickness varies systematically with both design factors, so within this design, it could not be entered as a covariate without collinearity with the cells being compared. The conclusion is that the molecular-weight effects reported above are not explained by thickness in these data and that separating the two requires films cast to a matched thickness by controlling the cast volume per unit area instead the solution concentration.

This interpretation is consistent with the spectroscopic, diffraction and microscopy results but is not demonstrated by them. The practical implication is that, at a fixed quercetin loading, how the flavonoid is incorporated decides which molecular weight gives the fastest release, so the two variables must be chosen together rather than optimised one at a time. A comparable dependence of release kinetics on the state of the incorporated phenolic has been reported for ferulic acid derivatives in biopolymer films [52].

### 3.6. Limitations

Only one AFM image was acquired per formulation, so roughness is reported without between-sample uncertainty. Antioxidant activity and quercetin release were measured in different media and could not be related quantitatively. Film thickness, although measured for all twelve films, was not matched across the series: it spanned 0.048 to 0.128 mm (Table 6). It was also the least well-replicated measurement reported here. Only one film per formulation × molecular weight combination was gauged at five positions, so the two-way analysis of variance given in the previous version was withdrawn, and the variation could not be apportioned between the two factors. Release is therefore reported per gram of dry film rather than normalised to thickness. The chemical stability of quercetin in the release medium was not determined either. Quercetin degrades in neutral aqueous buffer on a timescale of hours to days [38,39], and because no stability control was run in parallel and no chromatographic separation of intact flavonoid from its degradation products was performed, the absorbance at 429 nm reports what remained intact at each reading rather than what was released. The tabulated amounts are accordingly lower bounds, and the absolute release figures should not be used as such; a parallel incubation of quercetin standards in film-free medium, read at the same times, is the minimum control needed and is recommended for any repetition of this experiment.

## 4. Conclusions

How quercetin is incorporated into a PVA film determines the physical state of the flavonoid and how the polymer grade governs its release. Pre-dissolving quercetin in ethanol before casting leaves no detectable crystalline phase at either 0.5 or 1.0 g L^−1^, according to both FT-IR and X-ray diffraction; adding the same 1.0 g L^−1^ as a powder produces the full crystalline band set and the four diagnostic diffraction reflections. Surface roughness increases with quercetin content: both 1.0 g L^−1^ formulations were rougher than the control at every molecular weight. The two formulations that shared a loading differed in roughness by as much as either differed from the control, and the non-crystalline was rougher. Therefore, the method of incorporation affected topography. The two 1.0 g L^−1^ formulations were the controlled pair in this work, differing in one variable, and the spectroscopic, diffraction and antioxidant results agreed.

Antioxidant activity and extractable phenolic content were governed mainly by the contacting medium, decreasing in the order ethanol 95% > ethanol 10% > acetic acid 3%, which follows the solubility of quercetin and implies that these films are most active in contact with fatty foods and least active with acidic ones. The low-molecular-weight films yielded the most extractable antioxidant in all three assays, the yields from the medium and high grades being lower and indistinguishable from each other. The controlled pair is also informative: in the fatty simulant, the dispersed form delivered 1.8 times the DPPH activity and 2.1 times the phenolic content of the crystalline form at the same loading, while in the two aqueous simulants, where the solubility of the medium rather than the dissolution of crystallites set the limit, the two were indistinguishable. Two independent assays thus confirm the structural conclusion and establish that a non-crystalline flavonoid is the more available one in a medium that can dissolve it. For an antioxidant packaging film, the most promising combination among those tested is the ethanol-dissolved series, which left no detectable crystalline phase and yielded the greater extractable activity in the fatty-food simulant; within it, the low-molecular-weight grade produced the highest extractable antioxidant and achieved the fastest release, at the cost of the roughest surface, whereas the medium and high grades gave smoother films and a flatter response.

## Figures and Tables

**Table 1:** FT-IR band positions (cm^−1^) of PVA–quercetin films.

Film	$M_{w}$	O–H Stretch	C–H Stretch	C=O/H–O–H	CH_2_ Bend	C–H Wag	C–O Stretch	(C–O)–C–OH	Quercetin C=C	1520 Band (AU)
F1	low	3271	2927	1645	1414	1345	1234	1077	n.d.	−0.011
	medium	3272	2926	1643	1414	1339	1235	1076	n.d.	−0.010
	high	3271	2927	1638	1415	1345	1234	1076	n.d.	−0.009
F2	low	3269	2930	1642	1417	1340	1236	1074	n.d.	−0.011
	medium	3270	2930	1646	1415	1337	1234	1077	n.d.	−0.011
	high	3270	2930	1645	1416	1340	1234	1076	n.d.	−0.012
F3	low	3260	2940	1651	1416	1318	1264	1086	n.d.	−0.016
	medium	3263	2940	1659	1410	1318	1259	1086	n.d.	−0.000
	high	3257	2940	1646	1415	1326	1275	1086	n.d.	−0.022
F4	low	3260	2906	1662	1407	1316	1258	1088	1520	+0.057
	medium	3243	2920	1660	1407	1315	1257	1088	1519	+0.054
	high	3256	2908	1661	1406	1315	1257	1087	1519	+0.030

n.d., not detected: the quercetin C=C band at 1520 cm^−1^ was not resolved.

**Table 2:** Antioxidant capacity and total phenolic content of film extracts in three food simulants. Mean ± standard deviation of three films cast and dried separately from a common solution, each read in triplicate. Letters within an assay denote groups that did not differ per Tukey’s test (α=0.05) across all 27 formulation × simulant × molecular weight combinations. S1: ethanol:water 10:90 *v*/*v*; S2: acetic acid:water 3:97 *v*/*v*; S3: ethanol:water 95:5 *v*/*v*. F2 and F3 carried quercetin pre-dissolved in ethanol at 0.5 and 1.0 g L^−1^; F4 carried the same 1.0 g L^−1^ added as a powder. TE: Trolox equivalents; GAE: gallic acid equivalents.

Simulant	$M_{w}$	ABTS (μmol TE g^−1^)			DPPH, 30 min (μmol TE g^−1^)			Total Phenolics (mg GAE 100 g^−1^)		
		F2	F3	F4	F2	F3	F4	F2	F3	F4
S1	low	1005 ± 987 *^b^*	1215 ± 877 *^a^*	2666 ± 230 *^a^*	565 ± 96 *^a^*	469 ± 77 *^b^*	788 ± 395 *^a^*	2752 ± 581 *^c^*	2045 ± 32 *^c^*	3559 ± 828 *^b^*
	medium	1394 ± 373 *^a^*	489 ± 180 *^b^*	404 ± 299 *^b^*	276 ± 27 *^b^*	316 ± 55 *^b^*	161 ± 52 *^c^*	1754 ± 284 *^c^*	2252 ± 296 *^c^*	845 ± 369 *^d^*
	high	1202 ± 637 *^a^*	2271 ± 1208 *^a^*	544 ± 116 *^b^*	326 ± 33 *^b^*	386 ± 36 *^b^*	140 ± 12 *^c^*	1923 ± 273 *^c^*	2607 ± 252 *^c^*	1344 ± 568 *^d^*
S2	low	766 ± 109 *^b^*	2263 ± 264 *^a^*	2701 ± 224 *^a^*	331 ± 29 *^b^*	403 ± 147 *^b^*	476 ± 158 *^b^*	1738 ± 206 *^c^*	2093 ± 636 *^c^*	3482 ± 739 *^b^*
	medium	597 ± 236 *^b^*	95 ± 61 *^b^*	979 ± 149 *^b^*	162 ± 15 *^c^*	212 ± 39 *^b^*	173 ± 15 *^c^*	796 ± 48 *^d^*	756 ± 412 *^d^*	759 ± 320 *^d^*
	high	242 ± 94 *^b^*	568 ± 259 *^b^*	30 ± 22 *^b^*	217 ± 14 *^b^*	253 ± 14 *^b^*	142 ± 6 *^c^*	341 ± 85 *^d^*	1367 ± 208 *^d^*	343 ± 112 *^d^*
S3	low	600 ± 194 *^b^*	827 ± 226 *^b^*	1067 ± 814 *^b^*	611 ± 131 *^a^*	615 ± 28 *^a^*	266 ± 71 *^b^*	4565 ± 657 *^b^*	5017 ± 374 *^b^*	1868 ± 799 *^c^*
	medium	987 ± 228 *^b^*	2310 ± 261 *^a^*	1165 ± 183 *^a^*	378 ± 108 *^b^*	694 ± 107 *^a^*	521 ± 140 *^b^*	3398 ± 1115 *^b^*	4893 ± 137 *^b^*	3236 ± 706 *^c^*
	high	1069 ± 197 *^b^*	1270 ± 612 *^a^*	1823 ± 217 *^a^*	387 ± 24 *^b^*	865 ± 52 *^a^*	392 ± 77 *^b^*	2772 ± 332 *^c^*	6717 ± 722 *^a^*	2842 ± 698 *^c^*

**Table 3:** Quercetin released into PBS from cast films under static conditions.

Time (h)	F2 (0.5 g L^−1^, Ethanol)			F3 (1.0 g L^−1^, Ethanol)			F4 (1.0 g L^−1^, Powder)		
	L	M	H	L	M	H	L	M	H
24	12.7 ± 1.5	25.5 ± 5.2	29.2 ± 5.0	32.4 ± 10.4	14.7 ± 6.4	15.1 ± 0.8	18.8 ± 0.9	21.2 ± 1.1	26.6 ± 5.2
96	14.6 ± 2.4	30.4 ± 4.9	30.5 ± 5.7	37.9 ± 12.3	19.0 ± 9.5	18.2 ± 0.8	24.0 ± 1.1	23.8 ± 3.0	30.9 ± 6.1
120	15.6 ± 2.7	32.5 ± 4.4	29.3 ± 3.3	35.6 ± 12.4	15.7 ± 9.6	15.9 ± 1.6	21.4 ± 1.9	22.5 ± 3.6	25.8 ± 5.5
192	30.3 ± 5.3	35.9 ± 5.7	31.4 ± 5.0	45.1 ± 13.8	21.6 ± 10.5	21.7 ± 3.7	27.8 ± 2.6	27.1 ± 1.3	31.3 ± 5.1
288	31.4 ± 2.8	46.1 ± 3.2	49.8 ± 7.6	57.6 ± 17.8	31.0 ± 17.5	30.8 ± 2.4	37.2 ± 0.7	43.6 ± 7.8	56.1 ± 15.9
Pooled	22.6	34.1	34.1	41.7	20.4	20.4	25.8	27.6	34.1

*General conditions:* PBS (pH 7.4, 25 °C, dark, static) from 0.5 × 0.5 cm specimens (exposed area of 0.25 cm^2^ per face) in 3 mL of buffer. Concentrations expressed in μmol per g of dry film. Values represent mean ± standard deviation (n=3 independent films), quantified at λ=429 nm using the calibration A=0.0023C−0.0215 ($R^{2}=0.9949$). The final row denotes the time-pooled mean. Evaluated with n=2 specimens: one F3 (medium molecular weight) specimen was lost prior to the first measurement, and one F2 (low-molecular-weight) specimen was unread at 24, 96, and 120 h.

**Table 4:** X-ray diffraction of all films.

Film	$M_{w}$	PVA (101) (2 θ, °)	FWHM (°)	Scherrer (nm)	Crystallinity (%)	Quercetin, Diagnostic	Quercetin, Other
F1 (PVA only)	low	19.75	3.32	2.43	31.1	0.019	0.034
	medium	19.50	2.62	3.08	33.0	0.044	0.029
	high	19.90	2.06	3.91	41.8	0.014	0.019
F2 (0.5 g L^−1^, ethanol)	low	20.05	2.11	3.82	37.7	0.014	0.016
	medium	20.00	2.16	3.73	36.8	0.019	0.011
	high	20.10	1.76	4.58	49.2	0.007	0.008
F3 (1.0 g L^−1^, ethanol)	low	19.95	2.94	2.75	36.1	0.045	0.069
	medium	20.39	1.57	5.13	62.9	0.019	0.014
	high	19.95	2.27	3.55	48.7	0.036	0.057
F4 (1.0 g L^−1^, powder)	low	19.85	3.22	2.51	32.6	0.918	0.099
	medium	19.85	2.21	3.65	39.0	0.971	0.072
	high	19.85	2.16	3.73	39.9	0.905	0.066

**Table 5:** Surface roughness of all films from 20 × 20 μm atomic force micrographs. $S_{a}$ is the arithmetic mean deviation, $S_{q}$ the root mean square deviation and $S_{z}$ the peak-to-valley height; the last two columns are the mean and standard deviation of $S_{q}$ over the four image quadrants.

Film	$M_{w}$	$S_{a}$ (nm)	$S_{q}$ (nm)	$S_{z}$ (nm)	$S_{q}$ Quadrant Mean (nm)	$S_{q}$ Quadrant SD (nm)
F1 (PVA only)	low	78.1	114.9	1310	103.8	22.2
	medium	52.4	64.1	257	28.2	22.0
	high	24.9	30.3	203	10.7	7.0
F2 (0.5 g L^−1^, ethanol)	low	91.4	114.5	689	44.4	31.6
	medium	29.0	37.4	385	35.6	7.0
	high	16.2	20.3	140	12.8	3.4
F3 (1.0 g L^−1^, ethanol)	low	1013.1	1211.0	6116	828.9	424.6
	medium	75.5	97.5	901	46.3	17.8
	high	253.4	312.4	1723	255.9	22.7
F4 (1.0 g L^−1^, powder)	low	115.9	143.7	1010	45.6	32.7
	medium	69.3	85.4	395	39.5	7.6
	high	79.4	99.2	616	62.3	26.4

**Table 6:** Thickness of PVA–quercetin films, mean ± standard deviation of five measurements per film.

Film	Low $M_{w}$	Medium $M_{w}$	High $M_{w}$
F1	0.086 ± 0.011	0.128 ± 0.013	0.080 ± 0.010
F2	0.098 ± 0.016	0.050 ± 0.010	0.048 ± 0.008
F3	0.126 ± 0.027	0.116 ± 0.005	0.072 ± 0.011
F4	0.068 ± 0.033	0.090 ± 0.007	0.080 ± 0.012

Thickness in mm.

## Data Availability

The raw antioxidant assay data, the native AFM height files and the scripts used for the statistical analysis and for computing the roughness parameters are available from the corresponding author on request.
